# Tarsal tunnel syndrome: are we really investigating vascular causes adequately in clinical practice?

**DOI:** 10.1590/0100-3984.2025.58.e7-en

**Published:** 2025-11-29

**Authors:** Marcelo Rocha Corrêa da Silva

**Affiliations:** 1 Email: rochacorreasilva@hotmail.com. MD, Specialist in Diagnostic Imaging and Coordinator of the General Ultrasonography Sector, Hospital Israelita Albert Einstein, São Paulo, SP, Brazil. Email: rochacorreasilva@hotmail.com

Tarsal tunnel syndrome (TTS) results from compression of the tibial nerve or its branches within the tarsal tunnel. It is a clinical condition with a low (0.4–0.5%) population prevalence^**1**^, and its symptoms are broad and nonspecific. The absence of well-established diagnostic criteria in the literature makes its diagnosis challenging, which can result in underdiagnosis or diagnostic errors^**2**^.

The diagnosis of TTS is essentially clinical, with imaging methods being used in order to identify a possible causal factor for the compression. According to Fantino et al.^**3**^, the cause can be identified in 60–80% of cases^**4**^. Conversely, the etiology is considered idiopathic in 20–40% of cases, with an idiopathic etiology being particularly prevalent among patients with diabetes.

Currently, it is essential to make an accurate diagnosis of TTS in order to implement appropriate clinical management, especially in cases requiring surgical treatment. It is known that clinical outcomes are directly related to knowledge of the etiological factors and are significantly better when the causal element is identified than when the etiology is idiopathic^**5**^.

In an article published in **Radiologia Brasileira**, Soares et al.^**6**^ demonstrated the accuracy of ultrasound in the diagnosis of TTS, highlighting its favorable attributes and inherent advantages: high spatial resolution; low cost compared with magnetic resonance imaging; wide availability; and the ability to obtain dynamic, comparative, and orthostatic images. Compared with magnetic resonance imaging, ultrasound offers better spatial resolution, an advantage that is especially relevant in the study of the tarsal tunnel—a small structure—allowing detailed visualization of the anatomical relationships that its components have with the tibial nerve and its branches.

Among the causes of compressive neuropathies of the tarsal tunnel, the most prominent are bony, myotendinous, expansile, traumatic, and vascular causes. However, there is still a lack of precise data in the literature on the prevalence of each etiopathogenic group. Within the spectrum of vascular anomalies, Soares et al.^**6**^ cite varicose veins, which constitute the most common vascular cause, reportedly accounting for 13–24% of cases^**7–9**^, as well as venous thrombosis, tortuosity of the posterior tibial artery, tibial vein aneurysm, and vascular malformations.

When investigating vascular causes, it is essential to identify extrinsic compression of the tibial nerve or its branches, thus avoiding false positives (incidental and clinically irrelevant findings). To that end, dynamic maneuvers and postural variations should be employed during the ultrasound examination.

Soares et al.^**6**^ highlighted the ability of ultrasound to accurately diagnose TTS, showing that it can facilitate the differential diagnosis with other conditions, such as peripheral polyneuropathy, Baxter’s neuropathy, and plantar fasciitis.

The Soares et al.^**6**^ article represents a valuable contribution for the general radiologist, reinforcing fundamental concepts about TTS and underscoring the importance of ultrasound in the current diagnostic context and in the clinical management of cases. Research involving ultrasound of peripheral nerves is a quite promising field that has been gaining increasing scientific relevance as the equipment employed achieves extremely high levels of resolution, allowing the investigation of a variety of clinical situations that, until recently, were beyond the reach of any imaging modality. This will result in patient care that is more timely and effective, as well as a better cost-benefit ratio for the health care system.
